# Fibrin clot characteristics and anticoagulant response in a SARS‐CoV‐2‐infected endothelial model

**DOI:** 10.1002/jha2.407

**Published:** 2022-03-22

**Authors:** Conor McCafferty, Leo Lee, Tengyi Cai, Slavica Praporski, Julian Stolper, Vasiliki Karlaftis, Chantal Attard, David Myint, Leeanne M. Carey, David W. Howells, Geoffrey A. Donnan, Stephen Davis, Henry Ma, Sheila Crewther, Vinh A. Nguyen, Suelyn Van Den Helm, Natasha Letunica, Ella Swaney, David Elliott, Kanta Subbarao, Vera Ignjatovic, Paul Monagle

**Affiliations:** ^1^ Department of Paediatrics The University of Melbourne Melbourne Victoria Australia; ^2^ Haematology Murdoch Children's Research Institute Melbourne Victoria Australia; ^3^ Department of Microbiology and Immunology The Peter Doherty Institute for Infection and Immunity The University of Melbourne Melbourne Victoria Australia; ^4^ Heart Regeneration Murdoch Children's Research Institute Melbourne Victoria Australia; ^5^ TA Scientific Pty. Ltd. Taren Point Sydney New South Wales Australia; ^6^ Department of Occupational Therapy Social Work and Social Policy La Trobe University Melbourne Victoria Australia; ^7^ Neurorehabilitation and Recovery Florey Institute of Neuroscience and Mental Health University of Melbourne Melbourne Victoria Australia; ^8^ Tasmanian School of Medicine University of Tasmania Hobart Tasmania Australia; ^9^ Melbourne Brain Centre Royal Melbourne Hospital and University of Melbourne Melbourne Victoria Australia; ^10^ Department of Neurology and Stroke Monash Health Hospital Melbourne Victoria Australia; ^11^ Department of Psychology and Counselling La Trobe University Melbourne Victoria Australia; ^12^ WHO Collaborating Centre for Reference and Research on Influenza The Peter Doherty Institute for Infection and Immunity Melbourne Victoria Australia; ^13^ Department of Clinical Haematology The Royal Children's Hospital Melbourne Victoria Australia; ^14^ Kids Cancer Centre Sydney Children's Hospital Randwick New South Wales Australia

**Keywords:** anticoagulation, blood coagulation, cell culture, electron microscopy, viruses

## Abstract

Coronavirus disease 2019 (COVID‐19) patients have increased thrombosis risk. With increasing age, there is an increase in COVID‐19 severity. Additionally, adults with a history of vasculopathy have the highest thrombotic risk in COVID‐19. The mechanisms of these clinical differences in risk remain unclear. Human umbilical vein endothelial cells (HUVECs) were infected with SARS‐CoV‐2, influenza A/Singapore/6/86 (H1N1) or mock‐infected prior to incubation with plasma from healthy children, healthy adults or vasculopathic adults. Fibrin on surface of cells was observed using scanning electron microscopy, and fibrin characteristics were quantified. This experiment was repeated in the presence of bivalirudin, defibrotide, low‐molecular‐weight‐heparin (LMWH) and unfractionated heparin (UFH). Fibrin formed on SARS‐CoV‐2 infected HUVECs was densely packed and contained more fibrin compared to mock‐infected cells. Fibrin generated from child plasma was the thicker than fibrin generated in vasculopathic adult plasma (*p* = 0.0165). Clot formation was inhibited by LMWH (0.5 U/ml) and UFH (0.1–0.7 U/ml). We show that in the context of the SARS‐CoV‐2 infection on an endothelial culture, plasma from vasculopathic adults produces fibrin clots with thinner fibrin, indicating that the plasma coagulation system may play a role in determining the thrombotic outcome of SARS‐CoV‐2 infection. Heparinoid anticoagulants were most effective at preventing clot formation.

## 1 INTRODUCTION

The coronavirus disease 2019 (COVID‐19) pandemic, caused by SARS‐CoV‐2, is linked to haemostatic dysregulation with both severe and mild cases of COVID‐19 associated with coagulopathy [1]. Between 10% and 20% of COVID‐19 patients are admitted to the intensive care unit, and 31% of those patients experience a thromboembolic event [2, 3]. Hospitalised COVID‐19 patients have increased fibrin monomer and d‐dimer levels, with severe and critical COVID‐19 patients having prolonged prothrombin time and thrombocytopenia [4, 5, 6, 7]. There is also substantial evidence that COVID‐19 may trigger vasculitis and vasculopathy, which may be linked to the systemic effects of severe disease [8]. COVID‐19‐associated coagulopathy contributes significantly to the overall morbidity and mortality from COVID‐19, with post‐mortem evidence of thrombosis detected in the lung vasculature [9]. COVID‐19‐associated coagulopathy does not impact all people in the same way. COVID‐19‐infected children younger than 12 years of age have low rates of thrombosis, while adults with vasculopathy and COVID‐19 have significantly higher thrombotic rates and suffer a rapid and more severe decline in health [10, 11].

While there is an abundance of research into COVID‐19 and the response to SARS‐CoV‐2, a comprehensive understanding of underlying mechanisms of COVID‐19‐induced coagulopathy is lacking. An overwhelming inflammatory response coupled with increased platelet activation, increased blood viscosity and endothelial injury create a prothrombotic vascular environment [12, 13, 14]. The close interaction of inflammation, immunity and coagulation has been well established, and their co‐functioning has been termed thromboinflammation [15]. Inflammatory molecules, endothelial activation or the pathogen itself may interrupt or stimulate coagulation at any stage of blood‐clotting [16]. Certain viral infections, such as Ebola, promote a bleeding phenotype, while other viral infections, such as Influenza A, promote thrombosis [17, 18]. Avian influenza A/H5N1 infection exemplifies the complex interplay in thromboinflammation, as this virus stimulates both a bleeding and thrombotic phenotype [19].

There are multiple factors that can affect fibrin characteristics in a blood clot, including changes in pH, temperature and surrounding ion concentration [20]. However, the impact of a viral infection on the fibrin clot structure is currently unknown. When considering potential interventions, it is crucial to understand the structure of the SARS‐CoV‐2‐induced blood‐clot, particularly as factors such as fibrin fibre thickness and density can predict clot stiffness, resistance to lysis and therefore disease‐related morbidity [21, 22, 23].

Anticoagulation is widely used in the treatment of severe COVID‐19; however there is some debate as to the optimal drug, dose or administration schedule [24]. Unfractionated heparin (UFH) and low‐molecular‐weight heparin (LMWH), bivalirudin and defibrotide are among the drugs that have been used and/or studied in the setting of COVID‐19 [25, 26, 27]. The heparinoids (UFH and LMWH) both act upon antithrombin to inactivate thrombin, FXa and FIXa. UFH is large enough to act by binding antithrombin and thrombin, while the smaller LMWH binds antithrombin and FXa directly [28]. Bivalirudin, a direct thrombin inhibitor, binds to thrombin to produce a transient inhibitory effect of coagulation [29]. The mechanism of anticoagulation of defibrotide is not fully understood, although some of its pathways of action include directly antagonising thrombin, inhibiting the tissue factor pathway, and up‐regulating tissue plasminogen activator [30, 31]. There is little clarity on the effectiveness of prophylactic anticoagulation, despite some evidence that prophylactic heparinoids may reduce mortality in hospitalised COVID‐19 patients [32]. Reliable evidence‐based interventions are required [33].

We hypothesised that SARS‐CoV‐2 infection of endothelial cells promotes thrombosis and that this could be simulated in vitro. Based on the evidence of vasculitis in the pathogenesis of COVID‐19, we further hypothesised that individuals with a pre‐existing vasculopathy may be at a higher risk. We aimed to show the impacts of SARS‐CoV‐2 infection on the fibrin characteristics in healthy children, adults and adults with vasculopathy and to study the impacts of anticoagulants on this system. To facilitate this, we also aimed to develop an in vitro endothelial cell‐culture model to examine the contribution of the endothelium to haemostasis.

## 2 METHODS

### 2.1 Cell culture

Human umbilical vein endothelial cells (HUVECs) were cultured in this experiment. To produce HUVECs, flasks were coated with Geltrex (Thermo Scientific A1413302) in Dulbecco's Modified Eagle Medium (DMEM) (Thermo Scientific, 111965118) in a ratio of 1:100, 1 h prior to seeding. HUVECs were cultured to 95% confluency and split on a 24 well Geltrex coated plate (0.05*10^6^) 2 days prior to the experiment. All cells were cultured in endothelial growth media‐2 (EGM‐2) (Lonza, CC3202).

### 2.2 Plasma sample collection

This study was ethically approved under human research and ethics committee references: 34183, 32287 and H2010/03588‐amendment: 21225, SSA/11/MH/104 and AM/21225/Austin‐2020‐211015(v2). Blood from healthy children was collected as a part of the HAPPI Kids study [34]. Specifically, healthy children attending the hospital for minor, elective day‐surgery were enrolled into the study. Blood was collected via the cannula inserted as a part of standard clinical care. Blood from healthy adults was collected by venepuncture. Blood samples were collected in tubes containing 1‐part sodium citrate to 9‐parts blood. Plasma was collected from blood samples by centrifugation at 2500 g for 10 min and stored at −80°C until experimentation. Vasculopathic plasma samples were collected from adults who had previously suffered from their first ischaemic stroke episode and were enrolled in the START study [35]. Blood was collected from vasculopathic adults by venepuncture of the antecubital region tubes containing 1‐part sodium citrate to 9‐parts blood. Plasma was extracted by centrifugation at 1100–1300 g for 10 min and stored at −80°C until experimentation.

Plasma samples from children, adults and vasculopathic adults were all collected as part of studies undertaken prior to the COVID‐19 pandemic. Hence all samples were from SARS‐CoV‐2 negative subjects. Plasma was thawed and formed into healthy child, healthy adults and vasculopathic adult plasma pools on the day of each experiment. After formation, plasma pools were stored at 4°C and used within 4 h of thawing.

### 2.3 Viral infection

The SARS‐CoV‐2 virus (Vic/01) was isolated in Vero cells at the Peter Doherty Institute as previously described [36]. Influenza A/Singapore/6/86 (H1N1) virus was obtained from the WHO Collaborating Centre for Reference and Research on Influenza (Melbourne) and was used as a non‐coronavirus control. Cell culture infections using SARS‐CoV‐2 were performed at Biosafety level 3 laboratory conditions. Viruses were diluted in phosphate‐buffered saline (PBS) to achieve a viral inoculum of 200 µl/well at 0.1–1 multiplicity of infection (MOI). Cells were gently washed in PBS before addition of virus. PBS was used as a control in mock‐infected cells. Inoculated cells were incubated at 37°C and 5% CO_2_ for 1 h, after which the inoculum was removed by pipetting. Cells were then washed with PBS and incubated in the appropriate culture medium at 37°C and 5% CO_2_.

This viral infection procedure was repeated using Vero cells, with the supernatant used as a positive control for the viral replication detection experiment.

### 2.4 Clot formation experiment

HUVEC monolayers were cultured in 24‐well plates. Cells were inoculated with influenza A/Singapore/6/86 at 1.0 MOI and incubated for 48 h. Plasma was added to wells and clot formation triggered by the addition of 160 mM of calcium chloride. The formation of fibrin clots on the surface of cell monolayers was assessed to select the appropriate cell‐type for further experiments.

HUVECs were cultured and infected with influenza A/Singapore/6/86 (1.0 MOI) or SARS‐CoV‐2 (0.1 MOI) in 24‐well plates. HUVECs were incubated in EGM‐2 media for 46 h before incubation with recalcified plasma for 2 h. Before addition of plasma, cell supernatants were sampled for virus detection. Plasma was recalcified using 160 mM calcium chloride to overcome the effects of sodium citrate used to anticoagulated blood samples upon collection, allowing the coagulation system to proceed. This minimal concentration of calcium chloride was selected to balance the counteracting of the sodium citrate without excessively promoting thrombosis. Control wells were incubated with EGM‐2 instead of plasma. Following 2 h of incubation, cells were fixed with 2.5% glutaraldehyde. This experiment was performed in triplicate, with new plasma pools generated for each repeat.

### 2.5 Anticoagulation experiments

HUVECs were infected with SARS‐CoV‐2 as above. Prior to addition to the infected HUVECs, plasma pools were incubated with one of the following anticoagulation drugs: 0.02 µg/µl defibrotide, 0.005 µg/µl bivalirudin, 1 U/ml or 0.5 U/ml LMWH and 0.7 U/ml or 0.4 U/ml UFH. The doses of LMWH and UFH, representative of a higher therapeutic dose and lower prophylactic dose, were 0.25 U/ml and 0.125 U/ml of UFH, and 0.2 U/ml and 0.1 U/ml of LMWH.

### 2.6 Viral detection in HUVEC cultures

HUVECs were cultured on Nunc Thermanox coverslips (Thermo Fisher Scientific) in 24‐well plates and inoculated with Vic/01 or PBS (mock) at 0.1 MOI for 1 h. Supernatant samples were collected immediately after the inoculum was replaced with culture medium (0 h) and then at 24, 48 and 72 h from separate plates. The replication kinetics of SARS‐CoV‐2 in HUVEC cells was assessed by real‐time quantitative reverse‐transcription polymerase chain reaction (qRT‐PCR) of supernatant samples.

Viral RNA was extracted from supernatant fluid samples (HUVEC and Vero cells) using the QIAamp viral RNA mini kit (Qiagen) according to the manufacturer's instructions. Reverse‐transcription PCR was performed using the SensiFast Probe No‐ROX One‐Step Kit (Bioline) according to the following 20 µl reaction mix: 5 µl of sample RNA, 10 µl of 2x SensiFast Probe No‐ROX One‐Step reaction mix, 0.2 µl of reverse transcriptase, 0.4 µl of RiboSafe RNase inhibitor, 0.8 µl of 10 µM forward primer (E_Sarbeco_F1: 5′‐ACAGGTACGTTAATAGTTAATAGCGT‐3′), 0.8 µl of 10µM reverse primer (E_Sarbeco_R2: 5′‐ATATTGCAGCAGTACGCACACA‐3′), 0.08 µl of 25µM probe (E_Sarbeco_P1_FAM: 5′‐ACACTAGCCATCCTTACTGCGCTTCG‐3′) and 2.72 µl of water, under the following cycling conditions: 10 min at 55°C, one cycle; 3 min at 94°C, 1 cycle; 94°C for 15 s and 58°C for 30 s, 44 cycles. Amplification of the targeted gene segment is indicated by cycle threshold (C_t_) values, where values decreasing over time indicate replication and increasing viral load. The lower limit of detection was called at C_t_ values of 40 and above.

In each HUVEC clot formation experimental replicate, 400 µl of supernatant was sampled from two SARS‐CoV‐2‐infected wells and one mock‐infected well that did not receive human plasma at 48 h post‐inoculation. Sample aliquots were frozen at −80°C until testing by qRT‐PCR as described above.

### 2.7 Scanning electron microscopy

Gradient dehydration of the samples was carried out prior to performing sputter coating with gold/palladium for Scanning Electron Microscopy (SEM) imaging. The samples were treated with ethanol solutions of increasing concentrations in a stepwise manner as follows: 70% and 90% ethanol for 30 min each and 100% twice, for 60 min each.

The dehydration reagents (1 ml) were dispensed in each well containing the fixed samples. The plates were covered and allowed to rest at room temperature between reagent changes. At the completion of the treatment, ethanol was decanted and then drained by resting upside down on three plies of filter paper for 30 min. Complete removal of ethanol was achieved by evaporation for 3 h under low pressure (3 × 10^–3^ mbar). Evaporation was carried out in the chamber of a Quorum SC7620 sputter coater (Quorum Technologies, United Kingdom).

Using a small, pointed blade, the dried samples were gently cut off from the wells, which are released as thin films. They were immobilised on 12‐mm SEM stubs prepared with double‐sided carbon adhesives and then sputter coated with 2–3 nm of Gold/Palladium (Au/Pd).

Imaging was carried out using the backscatter electron detector of a Phenom XLII (ATA Scientific, Taren Point, Australia). The applied acceleration voltage was 10 kV and the spot size 3.3.

### 2.8 Image quantification

Fibrin strands in SEM images were manually quantified using FIJI ImageJ software (National Institutes of Health, NIH). Using the line and measure tool, two blinded independent reviewers selected 20 strands, from three randomly selected regions of the image (60 total strands total per image).

### 2.9 Statistical analysis

Analysis of image quantification was performed in Prism version 8.0.0 (GraphPad Software, CA, USA). Analysis of Variance (ANOVA) with Tukey's multiple comparisons test was performed between infection of the cells (mock‐infected, influenza A/Singapore/8/86 infected and SARS‐CoV‐2 infected HUVECs).

## 3 RESULTS

### 3.1 Viral detection in HUVEC cultures

**FIGURE 1:**
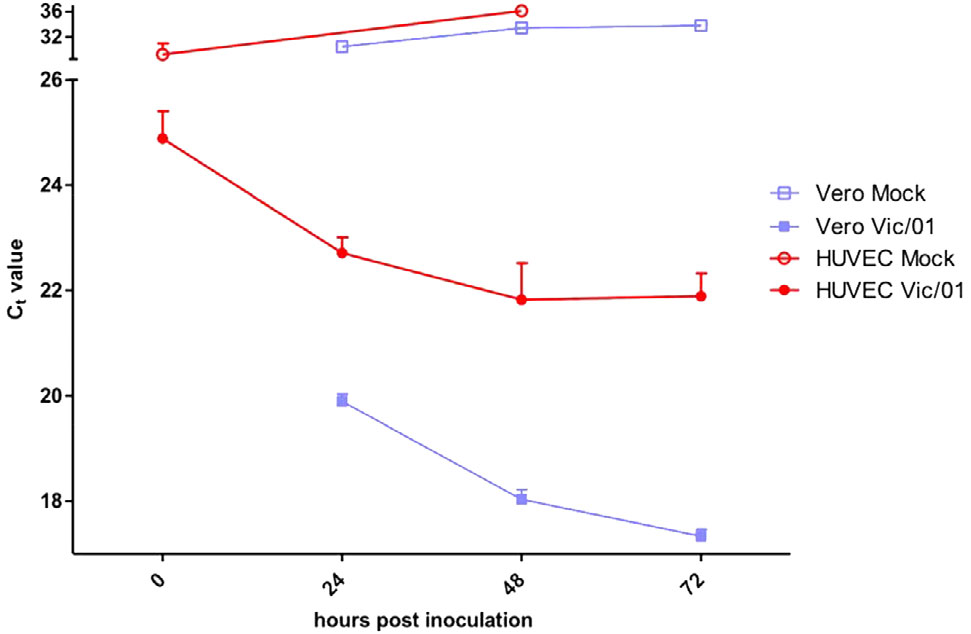
SARS‐CoV‐2 (Vic/01) E gene amplification from cell culture supernatant over time. Cell culture supernatants were collected from human umbilical vein endothelial cell (HUVEC) monolayers (red points) following inoculation with Vic/01 at 0.1 multiplicity of infection (MOI) (solid) or mock phosphate‐buffered saline (PBS) (clear). Reduction over time for Ct values indicates increasing viral load due to replication. Replication kinetics for HUVECs are compared to Vero cell monolayers (blue points)

In the replication kinetics experiment, amplification of the SARS‐CoV‐2 E gene was detected in HUVEC culture supernatant by qRT‐PCR following inoculation with Vic/01 at 0.1 MOI. Figure **1** shows the viral load increasing (C_t_ values reducing) from 0 h to 48 h in SARS‐CoV‐2 wells, indicating viral replication over time (solid red points). This trend is similar to the increase in viral load over time observed in SARS‐CoV‐2‐infected Vero cell monolayers (solid blue points). In contrast, no viral replication was detected in mock well supernatants over time. For each clot formation experiment, we confirmed the amplification of SARS‐CoV‐2 E gene from supernatant samples compared to mock control wells at 48 h post‐infection.

### 3.2 Clot formation experiment

Infection of HUVEC monolayers using influenza A/Singapore/6/86 (H1N1) has been previously reported to induce procoagulant activity in vitro [37]. HUVECs remained adherent to the wells at 48 h following inoculation and allowed for stable clot formation suitable for further imaging. HUVECs were therefore used in subsequent experiments using SARS‐CoV‐2.

**FIGURE 2:**
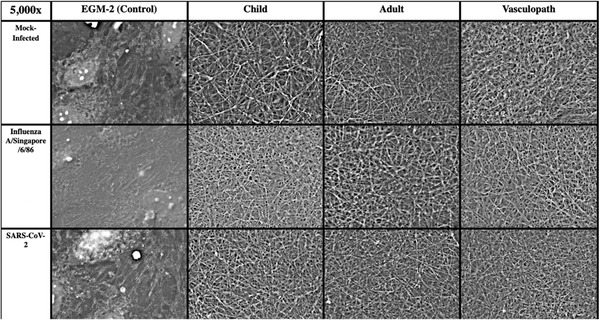
Representative scanning electron microscopy Images. Surface images of fibrin on cultured human umbilical vein endothelial cells (HUVECs) that have been incubated with human plasma. Conditions top‐to‐bottom are healthy HUVECs, influenza A/Singapore/6/86‐infected HUVECs and SARS‐CoV‐2‐infected HUVECs. Conditions left to right are incubated with endothelial growth media‐2 (EGM‐2) (control), healthy child plasma, healthy adult plasma and vasculopathic adult plasma. All images are presented at 5000× magnification

**FIGURE 3:**
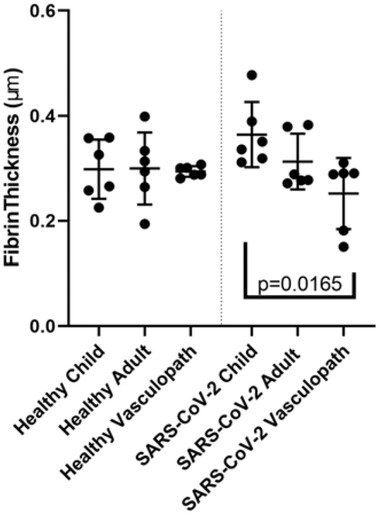
Fibrin characteristics of scanning electron microscopy images. Quantification of fibrin fibre thickness on healthy or SARS‐CoV‐2‐infected cells. X‐axis represents source of plasma (child, adult, vasculopath) and shows healthy cells on the left pane, and SARSCoV‐2‐infected cells on the right. Data points represent a mean fibrin strand thickness for three biological repeats, reviewed by two independent reviewers. Statistically significant differences

The clot formation experiment was repeated three times, with three different plasma pools. In the SARS‐CoV‐2 experiments, fibrin was visible in all wells that were incubated with plasma. None of the wells that were incubated with EGM‐2 alone showed any fibrin, and cells were present on wells after SARS‐CoV‐2 or influenza infection (Figure **2**). Fibrin was not detected in wells that only contained media, and there were no significant differences between fibrin in mock‐infected wells with different plasma. Fibrin density appeared to be greater in SARS‐CoV‐2‐infected cells compared to influenza and healthy controls, although there was no apparent difference in density of fibrin formed from plasma collected from healthy children, healthy adults or vasculopathic adults. Statistically significant differences in fibrin thickness across groups were confirmed by ANOVA (*p* = 0.21), and fibrin was significantly thinner in vasculopath clots compared to paediatric clots (*p* = 0.0165) (Figure **3**).

### 3.3 Drug response in SARS‐CoV‐2‐infected cells

**FIGURE 4:**
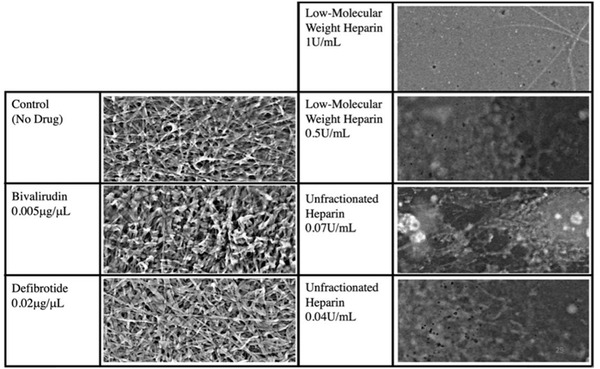
Anticoagulation experiment results. Comparison of anticoagulation effect or bivalirudin, defibrotide, low‐molecular weight heparin and unfractionated heparin. Representative images are selected from SARS‐CoV‐2‐infected human umbilical vein endothelial cells (HUVECs) incubated with vasculopathic adult plasma. Images were taken by scanning electron microscopy and are presented at 5000×

**FIGURE 5:**
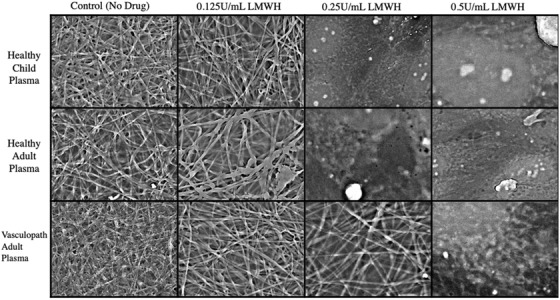
Low‐molecular weight heparin titration. Comparison of anticoagulation effect of low‐molecular weight heparin to 1/2 (0.25 U/ml) and 1/4 (0.125 U/ml) of the prophylactic dose (0.5 U/ml). Representative images are selected from SARSCoV‐2‐infected human umbilical vein endothelial cells (HUVECs) incubated with healthy child, healthy adult or vasculopathic adult plasma. Images are captured using scanning electron microscopy and presented at 5000× magnification

**FIGURE 6:**
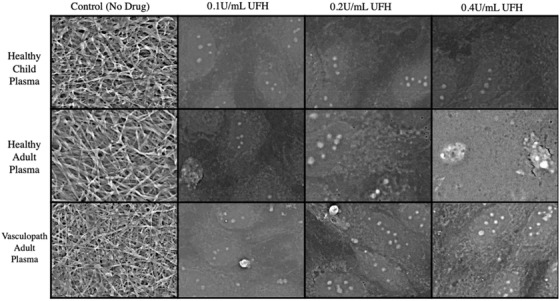
Unfractionated heparin titration. Comparison of anticoagulation effect of unfractionated heparin to 1/2 (0.2 U/ml) and 1/4 (0.1 U/ml) of the prophylactic dose (0.4 U/ml). Representative images are selected from SARS‐CoV‐2‐infected human umbilical vein endothelial cells (HUVECs) incubated with healthy child, healthy adult or vasculopathic adult plasma. Images are captured using scanning electron microscopy and presented at 5000× magnification

At standard concentrations, bivalirudin and defibrotide had no effect on preventing fibrin clot formation (Figure **4**) in SARS‐CoV‐2 infected cells. However, fibrin clot formation was prevented with prophylactic and therapeutic doses of LMWH and UFH. One quarter of the prophylactic doses of LMWH was effective in preventing fibrin formation in the presence of plasma from healthy children and healthy adults, but not vasculopathic adults (Figure **5**). One quarter of the prophylactic dose of UFH was sufficient to prevent fibrin formation in SARS‐CoV‐2‐infected cells across all plasma groups (Figure **6**).

## 4 DISCUSSION

We showed significant differences in fibrin characteristics in an in vitro model of SARS‐CoV‐2 infection of endothelial cells using plasma samples from healthy children, healthy adults and vasculopathic adults. Additionally, we provided important evidence that supports the use of heparinoids, particularly UFH, to prevent COVID‐19‐associated coagulopathy. Our model provided an endothelial cell surface that could be infected with two important viral pathogens (SARS‐CoV‐2 and influenza) and enabled examination of differences in clotting and responsiveness to antithrombotic therapies. While little is known regarding the pro‐coagulant effects of other viral pathogens, we believe this understanding of SARS‐CoV‐2 is crucial and sets the foundation for future studies of coagulation in infection scenarios. There is substantial evidence that SARS‐CoV‐2 can sufficiently infect endothelial cells both in vitro and in vivo [38]. Additionally, SARS‐CoV‐2 can lead to development of vasculitis in patients [8]. This model may be developed further to understand the relationships of endothelial cells, plasma and cellular elements in the blood, their response to different pathogens and the scope to manipulate those responses with different treatments.

### 4.1 Fibrin clot characteristics

The characteristics of a fibrin clot can be used as an indicator of clot strength, severity and resistance to lysis [20]. The size of the pores in the fibrin scaffold represents the clots strength, permeability and susceptibility to breakdown by lytic enzymes [39]. Increased thickness of fibrin fibres has been correlated with a less densely packed clot structure, that is, less resistant to lysis [40]. In the context of our results, this knowledge suggests that the thinner fibres produced in vasculopath plasma may represent a clot that has a greater resistance to natural fibrinolysis and is more likely to become pathological. Recent research has indicated that fibrinolysis is inhibited in patients with severe COVID‐19 [41]. This is particularly concerning in the context of our findings that formation of vasculopathic clots was also resistant to anticoagulation, particularly low doses of UFH. We expected that plasma from healthy children would produce thicker fibrin strands than healthy adults, and vasculopathic adults, as COVID‐19‐associated coagulopathy is extremely rare in children [42]. Based on our findings, we propose that individuals with a history of vasculopathy (such as stroke) are more vulnerable to serious COVID‐19‐associated coagulopathy.

### 4.2 Drug responses

Despite knowing that coagulopathy is a major complication of SARS‐CoV‐2 infection in humans since the virus pandemic emerged in early 2020, optimal treatment for COVID‐19‐induced coagulation has not been identified [24]. Our findings that LMWH and UFH were effective in preventing fibrin clot formation are particularly interesting. LMWH is often seen as a more attractive alternative to UFH due to more predictable pharmacodynamics, longer half‐life and less requirement for laboratory monitoring [43]. Our results suggest that UFH may be more effective in the context of COVID‐19. LMWH has a predominant anti‐Xa effect, while UFH has both anti‐Xa and anti‐IIa effect, all mediated via antithrombin. While this appears to suggest that the anti‐IIa behaviour of UFH is indeed the explanation for its increased effectiveness, we showed no anticoagulant effect of the direct Factor IIa inhibitor bivalirudin at therapeutic dose. However, the broader activity of UFH may be important. The viral replication data demonstrated that the difference was not due to a viricidal effect of UFH, which was unlikely in any case following the short duration of UFH incubation with the cell surfaces. However, we cannot exclude that UFH had an impact on endothelial cell drivers of plasma coagulation. While defibrotide interacts with a number of systems, it has been identified as an anticoagulant and profibrinolytic [31]. However, we failed to detect an effect of defibrotide on prevention of COVID‐19‐associated coagulation at therapeutic dose. While it may be possible that higher doses of bivalirudin or defibrotide may be effective, we did not go increase concentration beyond therapeutic dose.

It has been previously suggested, based on the structural differences, that paediatric clots were more susceptible to lysis [21]. We found no difference in the effectiveness of anticoagulants in children and healthy adults. This may suggest that the structural differences detected previously do not directly translate to clot behaviour, as previously hypothesised.

### 4.3 Endothelial haemostasis assay

Previous experiments have examined fibrin clot structure using SEM and implemented automated analysis software to characterise fibrin characteristics [21]. Previous studies formed clots on artificial surfaces such as filter paper, which may have been prothrombotic and did not account for the substantial impact of endothelial cells to coagulation [44]. Importantly, while we re‐calcified the citrated plasma samples using a small amount of calcium to reverse the effects of sodium citrate we did not add any specific coagulation activator—rather, the endothelial surface provided the stimulus for thrombosis.

The technique that we established for this study is versatile and modular, with applications for all haemostasis research. While we used SARS‐CoV‐2 infection, future studies may opt for different pathogens of interest. Similarly, we used a constant source of epithelial cells, but that could be varied in the setting of a constant plasma source, to examine differences in samples from, diabetic or vasculopathic cell lines or patients.

### 4.4 Limitations and next steps

HUVECs are derived from discarded umbilical cords of healthy babies and are typically used to model the behaviour of endothelial cells in vitro [45]. HUVECs do not necessarily represent all endothelial cell types in the body, and endothelial cells from differing sources may respond differently in vitro, particularly to infection [46]. Future studies may choose to focus on lung‐specific endothelial cells to mimic the site of infection more accurately. In this study, we studied the impact of anticoagulants; however future experiments that incorporate fibrinolytic drugs, such as tissue plasminogen activator or urokinase, after clot‐formation may be valuable to characterise fibrin‐clot resistance to fibrinolysis.

These experiments were conducted without blood cells, including red blood cells, white blood cells and platelets. This is of relevance, as UFH also acts on macrophages and platelets^,^ potentially masking some of the effects of the drug in our model [43]. Further, drugs were added 48 h following viral infection, and endothelial cells may have been activated significantly prior to addition of the drug. This may have reduced the effect of the anticoagulants, particularly defibrotide, which acts directly on the endothelium [30].

## 5 CONCLUSION

In conclusion, we demonstrated that fibrin characteristics caused by SARS‐CoV‐2 infection in endothelial cells exposed to plasma are impacted by age and prior vascular disease in the plasma source. Plasma from stroke patients produces fibrin clots in the presence of low doses of LMWH, while UFH was effective in all groups at extremely low doses, suggesting it may be valuable for COVID‐19‐associated coagulopathy.

## CONFLICT OF INTEREST

The authors declare that there is no conflict of interest that could be perceived as prejudicing the impartiality of the research reported.

## AUTHOR CONTRIBUTIONS

Samples were collected and coordinated by Conor McCafferty, Vasiliki Karlaftis, Chantal Attard, Leeanne M. Carey, David W. Howells, Geoffrey A. Donnan, Stephen Davis, Henry Ma, Sheila Crewther and Vinh A. Nguyen. Experiments were performed by Conor McCafferty, Leo Lee, Julian Stolper, David Elliott and David Myint. Data were analysed by Conor McCafferty, Leo Lee, Tengyi Cai and Slavica Praporski. Manuscript was written by Conor McCafferty and reviewed and revised by all authors. Kanta Subbarao, Vera Ignjatovic and Paul Monagle oversaw the study and manuscript.
